# Gastrointestinal stromal tumor

**DOI:** 10.1186/1477-7819-7-61

**Published:** 2009-08-01

**Authors:** Michael Stamatakos, Emmanouel Douzinas, Charikleia Stefanaki, Panagiotis Safioleas, Electra Polyzou, Georgia Levidou, Michael Safioleas

**Affiliations:** 1 4th Department of Surgery, University of Athens, School of Medicine, Attikon General Hospital, Athens, Greece; 2 3rd Department of Critical Care, Athens University, Eugenidion Hospital, Athens, Greece; 3 Department of Pathology, School of Medicine, University of Athens, Greece

## Abstract

**Background:**

GISTs are a subset of mesenchymal tumors and represent the most common mesenchymal neoplasms of GI tract. However, GIST is a recently recognized tumor entity and the literature on these stromal tumors has rapidly expanded.

**Methods:**

An extensive review of the literature was carried out in both online medical journals and through Athens University Medical library. An extensive literature search for papers published up to 2009 was performed, using as key words, GIST, Cajal's cells, treatment, Imatinib, KIT, review of each study were conducted, and data were abstracted.

**Results:**

GIST has recently been suggested that is originated from the multipotential mesenchymal stem cells. It is estimated that the incidence of GIST is approximately 10-20 per million people, per year.

**Conclusion:**

The clinical presentation of GIST is variable but the most usual symptoms include the presence of a mass or bleeding. Surgical resection of the local disease is the mainstay therapy. However, therapeutic agents, such as Imatinib have now been approved for the treatment of advanced GISTs and others, such as everolimus, rapamycin, heat shock protein 90 and IGF are in trial stage demonstrate promising results for the management of GISTs.

## Background

GISTs (Gastrointestinal tumors) are a subset of mesenchymal tumors and represent the most common mesenchymal neoplasms of GI (Gastrointestinal) tract. Gastrointestinal stromal tumors are KIT-expressing and KIT (tyrosine kinase receptor - CD117)-signaling driven mesenchymal tumors. Many GIST tumors have an activating mutation in either KIT or PDGFRα (Platelet-Derived Growth Factor Receptor Alpha) [1]. They account for <1% of all GI tumors. Their origin was at first attributed to Cajal's cells, in mesodermal tissue but it has nowadays been recognized that GISTs arise from multipotential mesenchymal stem cells [2]. However, GIST is a newly recognized tumor entity but the literature on these stromal tumors has swiftly expanded. In the past, these tumors were presumed to have elements of smooth muscle (smooth muscle origin), so they were classified as leiomyomas, leiomyosarcomas and leiomyoblastomas [3]. The term was first coined by Mazur and Clark, in 1983, in order to describe a heterogeneous group of gastrointestinal non-epithelial neoplasms. In 1998, Hirota reported that GISTs contained activating c-kit mutations, which play a central role in its pathogenesis [4]. Furthermore, GISTs express CD34 (cluster designation 34) and the KIT on their surface [5]. The origin of these tumors explicates their resistance to cancer chemotherapy. Moreover, it was their origin that lead to the introduction of a chemotherapeutic regimen, imatinib mesylate, a tyrosine kinase inhibitor for c-kit. GISTs are, finally, defined as pleomorphic mesenchymal tumors of the GI tract that express the KIT protein (CD 117- Protooncogene that encodes the transmembrane tyrosine kinase receptor CD 117 detected by flow cytometry in most cases of acute myeloid leukemia, in small numbers of T- and B-lymphoblastic lymphomas, and in some gastrointestinal stromal tumors - stem cell factor receptor) and often also CD34 (human progenitor cell antigen) on immunohistochemistry [6].

### Epidemiology

The incidence of GIST is estimated to be approximately 10-20 per million people, per year. Malignancy possibility is 20-30% [7-9]. However, the precise incidence of GIST is unknown because of the incomplete definition and classification [10]. Over 90% of GISTs occur in adults over 40 years old, in a median age of 63 years. However, GIST cases have been reported in all ages, including children. The incidence between the sexes is the same, although a study reported that there is a slight predominance of males [7]. There are no elements that indicate any association with geographic location, ethnicity, race or occupation. The most common location of GIST is stomach (50-60%) and small intestine (30%-40%). Five to ten percent of GISTs arise from the colon and rectum, and 5% are located in the esophagus. Other less common locations are those outside of the GI tract, like mesentery, retroperitoneum and omentum. However, there have been reported rare cases in the gallbladder, pancreas, liver and urinary bladder. In cases, where GIST occurs outside the GI tract, the tumors are known as extra - gastrointestinal stromal tumors (EGISTs) [11].

### Clinical Presentation

The clinical presentation of GIST is erratic. Furthermore, only 70% of the patients are symptomatic, while 20% are asymptomatic and 10% are detected at autopsy [6,7]. The symptoms and signs are not disease - specific and as a consequence, about 50% of GISTs have already metastases at the time of diagnosis. The clinical signs and symptoms are related to the presence of a mass or bleeding [12]. However, as it is mentioned above, 10% remain asymptomatic, because of their small size (< 2 cm) and they are diagnosed incidentally [13]. Bleeding comprises the most common symptom and it is attributed to the erosion of the gastrointestinal tract lumen. Bleeding occurring into the abdominal cavity leads to acute abdominal pain that usually ends up in emergency surgery. Nevertheless, bleeding can take place into the GI tract lumen, causing haematemesis, melena or anemia. Another common finding is the abdominal mass. However, most of the patients present with vague symptoms, such as nausea, vomiting, abdominal discomfort, weight loss or early satiety. Rapture of GISTs into the peritoneal cavity is rare and it causes life threatening intraperitoneal hemorrhage [14]. There are also symptoms related to the location of GIST. These symptoms include dysphagia in the esophagus, biliary obstruction around the ampula of Vater or even intrussusception, in the small bowel. Lymph nodes metastases are not common in GISTs. On the other hand, distant metastases most commonly occurs in GIST tumors of peritoneum, omentum, mesenteric areas and liver, while in EGISTs tumors are rare. At this point, it is important to mention that rectal GISTs frequently metastasize to the lung. GISTs have a high tendency to seed. The intra abdominal lesions result from tumor cell seeding into the abdominal cavity, whereas liver metastases derive probably from haematogenous spread. Finally, GIST patients may present with metastases in surgical scars [15].

### Diagnosis

GISTs show a variety of differentiation spectrum, ranging from fully differentiated tumors with myoid, neural or ganglionic plexus phenotype to those with incomplete or mixed differentiation. Nowadays, by the means of immunohistochemistry, it has become clear that the GIST cells are closely related to the multi-potential mesenchymal stem cells. In Table 1, differential diagnosis is being elucidated [16]. GISTs are positive for KIT [17]. Generally, GIST vary greatly in size from a few millimeters to >30 cm, the median size though is between 5 cm and 8 cm. Macroscopically, GIST usually has an exophytic growth and as a result, the intra-operative appearance commonly resembles of a mass, that is attached to the stomach, projecting into the abdominal cavity and displacing all the other organs [18]. Yet, mucosal ulceration is present in 50% cases. Additionally, GISTs are smooth gray and white tumors which are well circumscribed, usually with a pseudo-capsule. Less frequently, a small area of hemorrhage, cystic degeneration and necrosis may be visible [19]. GISTs have many different histological features. Gastric GISTs have a solid or nested form, often with a hyalinized stroma that shows myxoid change. GISTs in the small intestine, though, are more often spindled than epithelioid and may show a paragangliomatous pattern. Another characteristic is the eosinophilic structures, composed of collagen, which are stained brightly with PAS (periodic acid-Schiff stain). Even though, studies on esophageal, colonic and eGISTs are few, colonic and anorectal GISTs are more similar to intestinal than gastric GISTs, while esophageal GISTs resemble to gastric GISTs. Perinuclear vacuolization is a usual finding in gastric GISTs and reinforces the relationship between ICCs (Interstitial cell Cajal) and smooth muscle cells. Conversely, GISTs in the small intestine are more often spindled than epithelioid and may show a paragangliomatous pattern [12]. Diagnosis of GIST is often delayed, due to the vague nature of symptoms, for even 6 months after the onset of the symptoms. [10]. Although, the diagnostic procedure includes several examinations, like barium examination of the GI tract, computer tomography and angiography, none of them can establish the diagnosis. The preoperative percutaneous biopsy should not be used because it is associated with a significant risk of tumor rupture or dissemination [20]. The significance of endoscopic ultra-sound guided fine needle aspiration has been pointed out in several studies and the reported accuracy is 80% - 85% [21]. One recent study [22] suggested that EUS (Endoscopic ultrasound) findings on tumor characteristics, such as size (5 cm), irregular border, extraluminal growth, and heterogeneity can be used to predict malignant potential of GISTs. At this point, it should be emphasized that GISTs always have a malignant potential, although they may appear benign. One other study [23] evaluated pre-operative EUS criteria of 35 subepithelial upper gastrointestinal (UGI) lesions. Twenty six lesions were leiomyomas and 9 were leiomyosarcomas. This study was published in 1997, prior to the recognition of GIST as a distinct pathologic entity. In this study, tumor size (< 4 cm), irregular extraluminal border, echogenic foci and cystic spaces independently predicted malignant lesions. A French study similarly assessed EUS criteria of 56 surgically resected UGI (Upper GastroIntestinal) lesions and found that irregular extraluminal border, cystic spaces and malignant appearing lymph nodes were predictive of malignant or borderline stromal cell tumors [24]. Although EUS criteria are helpful in identifying GISTs, which should be resected, the key to pre-operatively determining malignant potential lies in cytology, histology, and immunohistochemistry. The development of EUS FNA as well as EUS trucut needle biopsy (TNB) has clearly improved endosonographers ability to diagnose GIST, but whether EUS FNA and EUS TNB can help determine malignant potential of GISTs pre-operatively is still unclear. Mitotic figures can be determined on EUS TNB specimens, but TNB specimens may not represent the entire lesion. There is considerable interest in performing immunohistochemistry on EUS FNA and EUS TNB specimens in attempt to predict malignant potential. For example, an abstract report of 17 patients with resected GISTs demonstrated that c-kit gene mutational analysis, as well as staining for MIB-1 were both predictive of malignant potential [25]. Another study demonstrated that the sensitivity and diagnostic yield of EUS-FNA for the diagnosis of GIST compare favorably with other well-accepted indications of this procedure, such as sampling pancreatic lesions and lymph nodes. More conventional sampling techniques, such as forceps biopsy or EMR, are limited in their clinical utility, given the difficulty of sampling lesions in a subepithelial location and the increased risk for perforation, respectively. In addition, a clear role for EUS-guided Tru-cut biopsy has yet to be defined, given inconsistent results in its ability to provide adequate tissue yield. More studies will have to be performed to further elucidate a well-defined role for these alternative sampling techniques. However, at present, EUS-FNA should be considered the procedure of choice to secure a tissue diagnosis of GIST. In examining features of GIST that are predictive of the ability to obtain adequate tissue yield, increasing size up to 10 cm, round/oval shape, and location in a specific sonographic wall layer were statistically significant in their ability to predict adequate tissue yield. Duodenal location, size ≥ 10 cm, irregular shape, and unclear sonographic wall layer were significantly associated with inadequate tissue yield and thus with non-diagnostic cytology samples. Statistical significance was demonstrated for each anatomic factor studied, while none of the biologic or procedural factors were found to be of significance. These findings demonstrate that EUS-guided FNA sampling may not necessarily be dependent on the histological composition of the tumor. There was a trend toward intermediate/high risk histological findings in the non-diagnostic group. However, this was likely because size represents 1 of the 2 criteria used in stratifying the malignant risk for GIST (mitotic count being the other), as described by Fletcher et al. [12]. The GISTs are CD117 positive in a percentage of 90% - 95%. Half of cases show cytoplasmic dot like positivity (Golgi pattern). Furthermore, dot like KIT immunoassaying is very suggestive of a c-kit mutation, being present in 71% of c-kit mutated GISTs [26].

**Table 1:** Tumor types in differential diagnosis with GIST

• leiomyoma
leiomyosarcoma (LMS)
• Schwannoma
malignant peripheral nerve sheath tumor (MPNST)
neurofibroma
• neuroendocrine tumor
carcinoid
carcinosarcoma
• fibromatosis or desmoid tumor
solitary fibrous tumor
inflammatory fibroid polyp
• angiosarcoma
clear cell sarcoma
liposarcoma
synovial sarcoma
• malignant mesothelioma
• dedifferentiated carcinoma
sarcomatoid carcinoma
• metastatic melanoma

### Prognostic Factors

GISTs have an uncertain clinical behavior ranging from benign to frankly malignant, making the outcome totally unpredictable. Over the years, many factors have been examined, such as size, histomorphology, immunohistochemistry and molecular genetics. However, it is difficult to predict the malignancy potential. Thus, there is not an accepted staging system for GIST. Multiple parameters have been considered as predictors of malignancy. At present, size and mitotic count appear to be the most useful predictors of the malignant behavior [26]. GISTs always have a malignant potential, even if they appear benign. Tumors <5 cm are usually low-risk, while those >5 cm are malignant. Even though size <5 cm is reassuring, we cannot always predict them as benign, as there is always the chance to metastasize [27]. Furthermore, the mitotic count is a reliable parameter. Mitoses <5 per 50 high power fields (HPF), usually characterizes GISTs as benign. Duodenal stromal tumors is characterized as benign have <2 mitoses per 50 HPF, while the cutoff for ileal GIST is 5 mitoses per 50 HPF. It is important to point out that fifty HPF is the minimum number of HPFs necessary to generate an accurate index of proliferative activity [28]. However, GISTs in stomach, measuring 5-10 cm, usually, have a good prognosis, as long as the mitotic count or Ki67 rate is low. On the other hand, small intestine tumors >5 cm behave in an aggressive way, regardless of the mitotic index. Finally, GISTs occurring anywhere, that measure >10 cm, tend to behave in a malignant way. Many studies [26] have indicated that there are several features, such as sclerosing that are related to a more favorable prognosis, while a hypercellular sarcomatous appearance predicts an aggressive behavior. In gastric tumors, diffuse nuclear atypia, coagulative necrosis and ulceration have been found to be prognostic unfavorable features while nuclear palisading and skeinoid fibers were favorable in a large series by Miettinen et al. [29]. Immunohistochemical markers may be of importance in predicting the malignant behavior of GISTs. Increased expression of cell cycle markers (MIB-I or Ki-67) have been linked to a less favorable prognosis in larger studies [30]. P16 is a tumor suppressor gene that inhibits cell cycling by arresting cells in G1 before entry into the S phase. P16 has been found to be down-regulated in malignant GISTs in some studies [31] but the same down-regulation has been found to be a prognostically favorable variable in other studies [32]. The National Institute of Health (NIH) Workshop, in 2001, suggested that a classification of GISTs in terms of their relative risk of aggressive behavior, rather than as benign or malignant, seems to be necessary. The guidelines recommend classifying GISTs into risk categories, based on size and mitotic count, emphasizing that no lesion can be definitely labeled as benign. Until recently, only mitoses and size of tumors were considered as highly important prognostic factors when evaluating the risk for metastasis and residual disease in patients with GISTs. The evaluation was based on a consensus approach after a GIST workshop by the National Institute of Health and often called the NIH Risk Stratification Categories. [12] Gastric tumors were later found to have a more favorable outcome than tumors arising from other locations [33] and the guidelines on risk classification have now been updated by Hornick and Fletcher [34] which includes location as an additional factor. Mutations in KIT exon 11 are found to be more common in larger tumors, and the presence of this mutation has been shown to have an adverse prognostic influence [35]. Deletions compared with point mutations in exon 11, have also been found to be a significant unfavorable factor in patients with gastric GISTs [36].

### Management of Gist

#### Management of localized Gist

Surgical resection of the local disease is the gold standard therapy. Its goal is complete resection of the disease with avoidance of tumor rupture [37]. Tumor size determines the survival and not the negative microscopic surgical margins [38]. Regional lymph node resection has no value since GIST rarely gives rise to lymph node metastases. However, the tumor size or its location may determine the exact extent of resection [39]. En block resection of the local disease is recommended when GISTs adheres to contiguous organ. GISTs are soft and fragile, so a tumor rupture must be avoided because it is associated with an increased risk for development of peritoneal implants [37]. Complete surgical resection is connected with 48-65% five year survival [39]. Partial resection must only be performed in case of large tumors, for palliative purposes or the control of symptoms or complications, such as compression of other organs, hemorrhage or even pain [40]. As it is already mentioned, surgery is the preferred management of GISTs, where feasible. However, there is also evidence that laparoscopic approach is effective, with minimal morbidity and no reported mortality [40]. If a laparoscopic resection is contemplated, several factors including patient characteristics, tumor size, location, invasion as well as the surgeon's experience need to be taken under consideration [41,42]. The aim of the laparoscopic surgery is the same, aiming at the complete removal of the tumor, avoiding tumor rupture, as peritoneal seeding affects disease free period [43,44].

#### Management of advanced GIST (metastatic and recurrent)

Standard treatment for primary gastrointestinal stromal tumor (GIST) is complete surgical resection, with the aim to obtain negative microscopic margins over the organ of origin [40]. In some cases, because of the anatomic site or the tumor size, complete resection is either not feasible or possible only through extensive procedures with expected major functional morbidity. Imatinib mesylate is a very active agent for tumor control in advanced and metastatic GIST [37]. GISTs have a high risk of metastatic relapse. The usual site of recurrence is the liver (65%), the peritoneal surface (50%) and both (20%). GIST's response to conventional chemotherapy is very poor (<10%), while radiotherapy is only used for analgesic purposes or in cases of intra peritoneal hemorrhage [26]. GISTs may show poor response to chemotherapy, but not to imatinib mesylate, also known as STI571 [11,45] which was found to act as a powerful selective inhibitor of tyrosine kinases of PDGFR and of c-kit receptor. Imatinib was initially designed as a PDGFR inhibitor and its efficacy as a tyrosine kinase was assessed in chronic myeloid leukemia [46]. The use of Imatinib mesylate in recurrent or metastatic, resectable or not GIST in prospective trial has shown response in 50% patients, and in approximately 75-85% patients have at least stable disease. The 2-year survival after Imatinib therapy is approximately 70% and 50% of the patients showed no progression of the disease [2]. Imatinib interruption after 1 year is associated with a high risk of relapse, even for patients in complete remission [47]. The treatment should continue until progression, intolerance or patient refusal. The treatment is usually well tolerated, but includes mild to moderate adverse effects such as edema (usually periorbital) [48], nausea, muscle cramps, diarrhea, headache, dermatitis, fatigue, vitiligo [49], hypothyroidism [50], cutaneous pigmentation [51] and abdominal pain. In patients with large bulky tumors, serious adverse events may include gastrointestinal, intraabdominal hemorrhages [52], cardiotoxicity [53] and serosal inflammation [54]. Other observed effects comprise neutropenia, leukopenia and abnormal liver function [55]. The ideal dose of Imatinib is not determined, but the current data show no added benefit with doses greater than 400 mg/day. All studies [44] on the dosage of Imatinib suggest that doses of 400-800 mg/day are safe, efficacious and patients tolerate it well. Imatinib was approved by the FDA for treatment of unresectable and metastatic GISTs on 1 February, 2002. Higher dosage is associated with symptoms of toxicity [46]. The common side effects of the drug consist of edema, rash, nausea, diarrhea, myalgia, fatigue, headache, and abdominal pain [56]. Recent study has confirmed that stopping of Imatinib is associated with an increased risk of disease progression but it is not known whether the discontinuation of Imatinib followed by reintroduction when the disease progresses is associated with a reduction in the survival [57]. Even though, Imatinib is a revolution for the management of GIST, it is not appropriate for all the cases of GIST. Even if it is rare, resistance to Imatinib has been reported [2,7,26]. There are patients, who do not respond to treatment with Imatinib or present an aggravation within 6 months during such treatment. These patients have primary resistance and usually have tumors with KIT exon 9 mutation or a non detectable kinase mutation [28]. Primary resistance to Imatinib is rare and affects only 15% of patients [39]. There is, also, another group of patients who has progression of tumor after at least 6 months of measurable response to Imatinib [55] and we used to say that they have a secondary resistance to Imatinib. Half of the patients, who initially respond, become resistant by 2 years after Imatinib initiation [57]. The common mechanism of acquired resistance is secondary kit mutation. Resistant lesions appear on imaging studies as a growing nodule in the pre-existing tumor. Primary and secondary resistance to Imatinib is also becoming a major clinical problem in the treatment of this disease. Therefore, new drugs that can be served as alternative therapies in Imatinib-resistant patients with GIST or that can be used in combination with Imatinib are needed [2]. The first clinical studies demonstrate that Imatinib is the first effective treatment for non resectable or metastatic GIST [56]. However, long-term results have not been extracted yet, because of the short time of use. It is obvious that further clinical studies must be designed [58-60].

#### Drugs for GISTs

The use of Imatinib as an adjuvant therapy after complete primary GIST resection is under evaluation. The American College of Surgeons Oncology Group (ACOSOG) has conducted a prospective trial to patients after complete resection of the tumor [42]. The dose of Imatinib was 400 mg/day for 12 months [26]. The data from this study showed promising results, since Imatinib [2] is well tolerated in the adjuvant setting. However, other trials [2,28], administering Imatinib and placebo as adjuvant therapy, showed no difference in the overall survival between the two groups. At present, the use of Imatinib in an adjuvant setting should be considered experimental and physicians should be encouraged to enroll patients in clinical trials. While tyrosine kinase inhibitors have improved survival in advanced GISTs, complete response is rare. Furthermore, it is now clear that the majority of patients who initially benefit from tyrosine kinase inhibitors eventually become resistant, with a median time to progression on Imatinib mesylate of 2 years. Responses to Imatinib GIST patients depend on the presence and genomic location of KIT mutations [1]. Furthermore, the use of Imatinib neoadjuvant therapy with or without an adjuvant treatment might help in controlling micrometastatic disease, since GIST tend to spread. The duration and dose of Imatinib in the neoadjuvant setting are yet undecided, however, less than 5% patients have complete clinical response to Imatinib. In patients who develop focal resistance, with some tumors progressing on Imatinib and others remaining stable [26], surgery can be considered for the progressive disease component. By resecting clones of disease that have acquired drug resistance, surgical debulking may prolong survival in patients with metastatic disease, as long as the remaining disease remains drug responsive [60]. In conclusion, the histological response to Imatinib is varied and does not correlate well with the clinical response. The clinical outcome in stable or partial responsive GIST patients does not seem to be influenced by either the duration of the Imatinib treatment, the histological response, or the size of the tumor. Second-site KIT mutations are rare in GISTs response to Imatinib, compared with Imatinib-resistant tumors, which harbor KIT kinase domain mutations in half of the cases. Chronic inhibition of KIT signaling by imatinib may induce tumor cells trans-differentiation into a smooth muscle phenotype, in a subset of cases, as suggested by the ultrastructural findings and microarray studies. Lastly, it is speculated that the presence of p53 gene alterations in GIST does not seem to affect clinical and histological response to imatinib [1].

The therapeutic effect of several compounds other than kinase inhibitors have been examined in models of GISTs. Rossi et al. [61] used knock-in mice with a *Kit *gain of-function mutation in the JM domain (Organoplatinum compound possessing antineoplastic activity) [62]. KIT-positive and imatinib-sensitive GISTs spontaneously developed in the knock-in mice. They administered an inhibitor of mTOR, RAD001 (everolimus), to the knock-in mice. Mammalian target of rapamycin (mTOR) regulates the translational response by phosphorylating components of the protein synthesis machinery. RAD001 did not induce apoptosis but near-complete arrest of cell-cycle progression in the imatinib-sensitive GISTs. Since phosphorylation of mTOR depends on KIT signaling in the imatinib-sensitive GISTs, RAD001 did not show any synergistic effect with imatinib in this setting. In contrast, RAD001 might be effective in imatinib-resistant GISTs [61]. Heat shock protein 90 (HSP90) protects KIT from proteasome-mediated degradation. Bauer et al [63] examined the effect of an HSP90 inhibitor (17-allylamino-18-demethoxy-geldanamycin, 17-AAG) on KIT-expressing and imatinib-sensitive, KIT-expressing but imatinib-resistant and KIT-non-expressing and imatinib-resistant human GIST cell lines. The proliferation of the KIT-expressing and imatinib-sensitive and KIT-expressing but imatinib-resistant cell lines was inhibited by 17-AAG, but that of the KIT-non-expressing and imatinib-resistant cell line was not. These results indicated that the expression of KIT is essential for the therapeutic effect of 17-AAG. Flavopiridol is a transcriptional repressor of numerous genes, including *Kit*. Sambol et al. 97 examined the effect of fl avopiridol on a KIT-expressing but imatinib resistant human GIST cell line. The flavopiridol treatment caused apoptosis of the target cells. These three compounds, RAD001, 17-AAG and flavopiridol, or their derivatives might be useful for treatment of imatinib-resistant GISTs. IGF1R is amplified and over-expressed in the majority of GISTs that lack c-KIT or PDGFRα mutations. More importantly, it has been shown, by a recent study that imatinib-sensitive and -resistant GIST cells respond equally well to a small molecular inhibitor of IGF1R, suggesting an alternative and/or complementary therapeutic regimen in the clinical management of GIST, especially in tumors that respond less favorably to imatinib-based therapy, including pediatric cases. These findings are particularly exciting given the number of agents targeting IGF1R that are currently being tested in clinical trials. It is feasible in the near future to initiate clinical trials by using IGF1R-targeted therapies for imatinib-refractory GIST patients, initially focusing on adult and pediatric GIST patients [64].

### Survival and Follow up

During the period of time that Imatinib did not been used for GIST therapy, the 5 year survival after the surgical resection was only 40-75%. The median survival of recurrent GIST after resection was 15 months in the pre-Imatinib era [60]. The prognosis of low risk GIST after complete resection was excellent, but the prognosis of high risk GIST was low and the rate of recurrence with 5 year survival ranged from 0% to 30%. However, after the introduction of molecular targeted therapy, Imatinib, there is a major improvement in the survival [65].

GISTs have an unpredictable behavior and a long term follow up is essential for all patients, independent of their benign or malignant characteristics. As the majority of those GISTs tends to recur within the first 3-5 years, intense follow up is required during this period [60]. According to the National Comprehensive Cancer Network guidelines, contrast CT (Computed Tomography Scan) of the abdomen and pelvis is recommended every 3-6 months for 3-5 years and then yearly. According to Novitsky et al. most of the recurrence occurs during the first 2 years after surgical resection [7]. They follow-up the patient with physical examination every 3-4 months for 2 years, then every 6 months for the next 2 years, then yearly. Chest X-ray and abdominal CT scan and blood test were obtained yearly [26]. Flexible upper endoscopy is performed at 6 months and 1-year postoperatively and then annually for 2 years. PET (positron emission tomography) scanning of abdomen, MR (Magnetic Resonance) imaging, or chest CT scan is done if abnormalities are found in any of the surveillance studies [28].

## Conclusion

**Figure 1:**
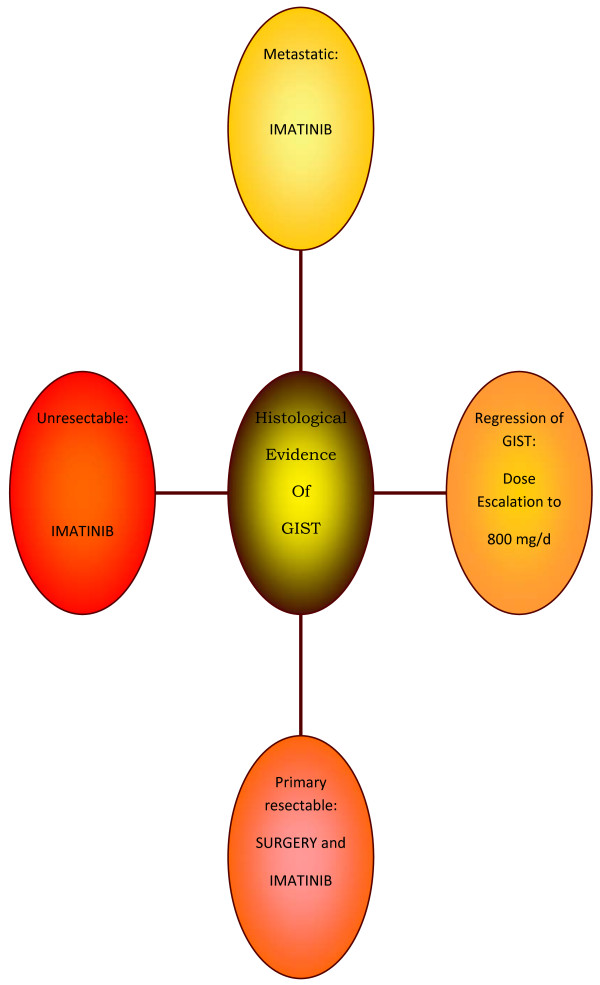
**Gist treatment**.

Gastrointestinal stromal tumors (GISTs) are the most common mesenchymal tumors of the GI system. In most cases, GISTs are characterized by gain-of-function mutations in the *KIT *proto-oncogene, most commonly involving exon 11, less frequently involving exon 9, and rarely involving exons 13 or 17 [66]. In GISTs without *KIT *mutations, gain-of-function mutations may occur in the platelet-derived growth factor receptor α (PDGFRα) gene, thereby providing an alternative oncogenic mechanism [67] GISTs' incidence, although rare, is on the rise, because of the improved diagnostic modalities, which offer accuracy [2]. The treatment of choice for primary GISTs remains complete surgical resection [20]. Imatinib mesylate is an oral tyrosine kinase inhibitor that has dramatically changed GIST therapy. This drug inhibits the KIT and PDGFRα tyrosine kinases as well as other members of the type III group of tyrosine kinases [68,69]. However, after the introduction of molecular targeted therapy with Imatinib, treatment of metastatic or recurrent GISTs is more effective and the survival rate has improved impressively [26]. Gastric GISTs are more common than small bowel GISTs. Patients with malignant gastric GISTs have a significantly better prognosis than patients with malignant small bowel GISTs. A statistically significant correlation was found between age and malignant potential of the GIST, by a recent study [70]. Though, the treatment of primary GIST is complete gross surgical resection, it is reasonable to consider to administer Imatinib mesylate as a preoperative therapy in localized bulky tumors, given its expected high response rate, as shown in a metastatic setting [5]. Surgical resection remains the mainstay of treatment. A formal indication to primary Imatinib in all cases of localized unresectable GIST had already been provided and it is consistently proposed by all available guidelines. Nevertheless the resectability of a tumor is hard to standardize and often considered surgeon dependent. It would, therefore, be rather difficult to list criteria universally acceptable for unresectable disease in order to provide formal indications to a preoperative treatment [71]. Figure 1 describes GIST treatment in revision. It is well known that the major predictive factor for tumor response to IM (Imatinib mesylate) therapy is mutational status. Ideally, it should be taken into account to select the patients who are more likely to benefit from the preoperative treatment. Nevertheless it may be difficult to assess at the decision time point. Moreover, tumor shrinkage could be observed even in cases with less favorable KIT/PDGFRα mutational status, with an overall median tumor reduction of 34%. Given the limited sample size, the extent of tumor shrinkage could not be correlated either to mutational status or to other clinical features, such as tumor site, initial tumor size, imatinib duration, and extent of pathologic response. In light of these results the presence of a less sensitive mutation like Exon 9 or of a wild-type KIT/PDGFRα mutational status should not be considered per se a contraindication to a preoperative treatment, the only exception being those point mutations with known complete resistance (i.e. D842V in Exon 18 on PDGRα gene). The only precaution is to strictly monitor the response, by early PET/TAC re-evaluation [72].

## Competing interests

The authors declare that they have no competing interests.

## Authors' contributions

MSt: search of the literature, partial English editing. ED: editing and correction. CS: editorship of the manuscript. PS: search of the literature. EP: editing. GL: histology consulting. MSa: final editing and corrections.
